# Algorithms for effective querying of compound graph-based pathway databases

**DOI:** 10.1186/1471-2105-10-376

**Published:** 2009-11-16

**Authors:** Ugur Dogrusoz, Ahmet Cetintas, Emek Demir, Ozgun Babur

**Affiliations:** 1 Center for Bioinformatics and Computer Engineering Dept., Bilkent University, Ankara, Turkey; 2 Computational Biology Center, Memorial Sloan-Kettering Cancer Center, New York, NY, USA

## Abstract

**Background:**

Graph-based pathway ontologies and databases are widely used to represent data about cellular processes. This representation makes it possible to programmatically integrate cellular networks and to investigate them using the well-understood concepts of graph theory in order to predict their structural and dynamic properties. An extension of this graph representation, namely hierarchically structured or compound graphs, in which a member of a biological network may recursively contain a sub-network of a somehow logically similar group of biological objects, provides many additional benefits for analysis of biological pathways, including reduction of complexity by decomposition into distinct components or modules. In this regard, it is essential to effectively query such integrated large compound networks to extract the sub-networks of interest with the help of efficient algorithms and software tools.

**Results:**

Towards this goal, we developed a querying framework, along with a number of graph-theoretic algorithms from simple neighborhood queries to shortest paths to feedback loops, that is applicable to all sorts of graph-based pathway databases, from PPIs (protein-protein interactions) to metabolic and signaling pathways. The framework is unique in that it can account for compound or nested structures and ubiquitous entities present in the pathway data. In addition, the queries may be related to each other through "AND" and "OR" operators, and can be recursively organized into a tree, in which the result of one query might be a source and/or target for another, to form more complex queries. The algorithms were implemented within the querying component of a new version of the software tool PATIKA*web *(Pathway Analysis Tool for Integration and Knowledge Acquisition) and have proven useful for answering a number of biologically significant questions for large graph-based pathway databases.

**Conclusion:**

The PATIKA Project Web site is http://www.patika.org. PATIKA*web *version 2.1 is available at http://web.patika.org.

## Background

Especially with the help of novel large-scale analysis methods, a massive amount of data is now being gathered on cellular processes [1-3]. Unfortunately, most of these data are fragmented and incomplete. One of the biggest challenges of bioinformatics today is to represent and integrate this type of knowledge effectively to construct a knowledge base that can act as a blueprint for simulations and other analysis methods, enabling us to better understand and predict the behavior of a cell [4].

Even though traditional way of representing cellular pathways with still images often yields very pretty pictures, such drawings are mostly not reusable. In addition, underlying ontology and notation are often far from being uniform or consistent, mostly dependent on implicit conventions rather than explicit, formal rules [4]. Recently, these problems have resulted in a major shift towards the use of more formal ontologies and to the dynamic representation of pathways that support programmatic integration and manipulation of pathways, regardless of the underlying ontology. Among these, graphs, one of the most common discrete mathematical structures [5], have been most popular for "in-silico" modeling of biological pathways, from metabolic pathways to gene regulatory networks to signaling pathways [4,6-8]. Such modeling is crucial for the field of systems biology, which deals with a systems-level understanding of biological networks. Three levels of increasing complexity are listed in [6] for the analysis of cellular networks, where network topology (global structural properties), interaction patterns (local structural connectivity), and network decomposition (hierarchical functional organization) are addressed at each level, respectively. Representing such complex networks as graphs makes it possible to investigate the topology and function of these networks using the well-understood concepts of graph theory and to predict their structural properties or to detect special structures or properties in them. In addition, this representation has made the systematic (i.e., programmatic) integration of these complex networks feasible. A comprehensive survey of such prediction, detection, and reconstruction methods can be found in [6].

Lately, an extension of this graph representation, namely *hierarchically structured graphs *or simply *compound graphs*, has become popular, in which a node of a biological network may recursively contain or include a sub-network of a somehow logically similar group of biological objects [4,9]. This extension provides many benefits for the analysis of biological pathways; most importantly, it reduces the complexity of large networks by decomposing them into distinct components or modules.

There has been much work on querying for the occurrences of sub-structures (from specified subgraphs to special sub-structures), such as graphlets or motifs in graph-based data, including pathways [6]. Most approaches employ a graph-matching algorithm to find one or all (exact or inexact) instances of the specified subgraph [10-12]. Others take a comparative approach to interpreting molecular networks, contrasting and aligning networks of different species and molecular types under varying conditions [13]. Graph algorithms, such as the shortest paths between a specified pair of objects in a graph database, have been in use for quite a while [14], whereas their use in graph-based pathway databases has only recently become popular [15-17].

**Figure 1:**
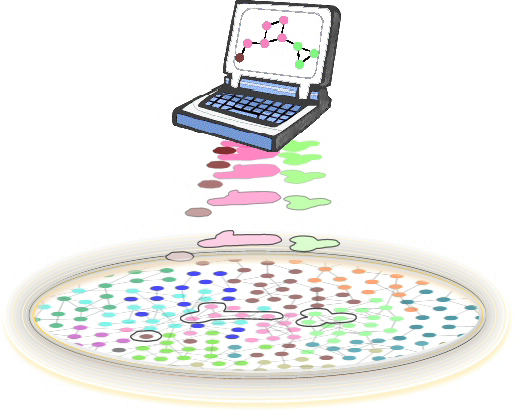
**How pathways are integrated**. Conceptual illustration of how pathways are integrated in a knowledge base (each pathway is colored distinctly), which is typically on disk, and how a sub-network of interest (parts of three different original pathways) may be extracted and displayed as a result of a query.

Here, we present a framework for querying a compound graph-based pathway database, as well as a number of graph-based queries and algorithms that are needed to implement these queries. We assume a database in which pathways are stored in an integrated manner, as opposed to a list of independent pathways. A query to the database is performed over this integrated, higher-level network of pathways, which aims to find a sub-network of interest (Figure 1). Such a query might require a rich set of graph algorithms. This framework and associated graph algorithms were implemented as part of a new version of the bioinformatics tool PATIKA*web *[18]. The range of graph-theoretic queries described as part of this framework is among the most comprehensive to be built so far and, in our best knowledge, is the first querying framework that accounts for *compound *structures (i.e., the grouping or abstractions of biological objects to an arbitrary level of depth) in a graph-based knowledge base.

## Definitions

Let *G *= (*V*, *E*) be a graph with a non-empty node set *V *and an edge set *E*. An edge, *e *= {*x*, *y*} or simply *xy*, joining nodes *x *and *y *is said to be *incident with *both *x *and *y*. Node *x *is called a *neighbor *of *y *and vice versa. A pathway graph *G *= (*V*, *E*) is a graph, where some of the edges in *E *are marked as *inhibition *edges (e.g., an interaction that disables or impedes the target reaction node via the source state node).

A path between two nodes *n*_0 _and *n*_*k *_is a non-empty graph *P *= (*V ', E'*) with *V' *= {*n*_0_, *n*_1_, ..., *n*_*k*_} and *E' *= {*n*_0_*n*_1_, *n*_1_*n*_2 _, ..., *n*_*k*-1_*n*_*k*_}, where *n*_*i *_are all distinct. *n*_0 _and *n*_*k *_are called the end points of path *P *= *n*_0_*n*_1 _... *n*_*k*_, whose *length*, denoted by |*P*| is the number of edges on it. A path is said to be *directed *if all its ordered edges are directed in the same direction. A directed path *P *is called an *incoming *(*outgoing*) path of node *n *if *P *ends at *target *(starts at *source*) node *n*. A directed path is called *positive *(*negative*) if it contains an *even *(*odd*) number of inhibitors (i.e., inhibition edges).

Given node sets *A *and *B*, an *A*-*B *path is a path with its ends in *A *and *B*, respectively, and no node of *P *other than its ends is from either set *A *or *B*. An *A*-path is a path where one of its end nodes is in *A*, and no other nodes and interactions are from *A*.

A path *C *with identical end nodes is called a cycle. A directed cycle is called *positive feedback *(*negative feedback*) if it contains an *even *(*odd*) number of inhibitors.

**Figure 2:**
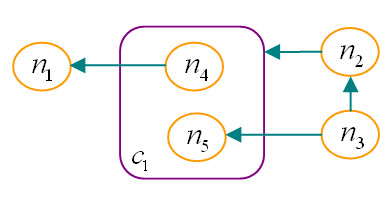
**Example compound graph**. An example compound graph (*V *(*G*) = {*n*_1_, *n*_2_, *n*_3_, *n*_4_, *n*_5_, *c*_1_}, *E*(*G*) = {*n*_2_*c*_1_, *n*_3_*n*_2_, *n*_3_*n*_5_, *n*_4_*n*_1_}, and *I *= {*c*_1_*n*_4_, *c*_1_*n*_5_}). The order, in which nodes are traversed, depends on the relationship between a compound structure and its members and on the relationship between members of a compound structure.

The *distance d*_*G*_(*x*, *y*), between two nodes *x *and *y *in graph *G*, is the length of a shortest *x*-*y *path in *G*. If *G' *= (*V ', E'*) is a subgraph of *G *= (*V*, *E*), and *G' *contains all the edges *xy *∈ *E *with *x*, *y *∈ *V'*, then *G' *is an *vertex-induced *or simply *induced *subgraph of *G*; we say that *V' *induces *G' *in *G *and write *G' *= *G *[*V'*]. If node *x *is the starting node of a directed path that ends up at node *y*, then node *y *is said to be in the *downstream *of node *x*; similarly, node *x *is said to be in the *upstream *of node *y*. A node *y *in the downstream of a node *x *is a *potential target *of *x*; similarly, *x *is a *potential regulator *of *y*. A *compound graph CG *= (*G*, *I*) is a 2-tuple of a graph *G *and a rooted directed *inclusion tree I *⊆ *V *× *V*, which defines nesting relations for *V *by partitioning it into *base nodes *(i.e., leaves of the inclusion tree) and *compound nodes *(Figure 2). Notice that we assume that edges of *G *cannot join two vertices, one of which is an ancestor of the other one in the inclusion tree.

### Ontology

The query framework and the algorithms described in the paper were all designed and implemented assuming the PATIKA ontology [4], which shows the utmost similarity to standard representations, such as BioPAX [19] and SBGN [20]. However, the results should be applicable to other graph-based pathway representations without difficulty.

**Figure 3:**
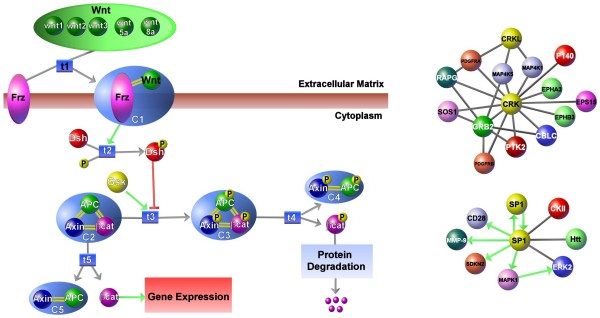
**Sample pathways represented by PATIKA ontology**. **(left) **Canonical wnt pathway containing examples of compound structures, such as regular abstractions (e.g., "protein degradation"), homology abstractions (e.g., 5 wnt genes), and molecular complexes (e.g., APC:Axin) [4]. **(right) **Partial human interaction networks containing PPIs and the transcriptional regulation interactions of proteins CRK and SP1, respectively.

The PATIKA ontology is based on a two-level qualitative model. At the entity level, interactions and relations can be addressed in an abstract manner, where the exact state or details of the involved entities is unknown, such as with protein-protein interactions, inferred relations, and literature-derived information. At the state/transition or mechanistic level, each entity is associated with a set of states that interact with each other via transitions. This level can capture more detailed information, such as compartments, molecular complexes, and different types of biological events (e.g., covalent modification, transportation, and association). This two-level representation elegantly covers most biological pathway-related phenomena, and is capable of integrating information present in the literature and in molecular biology databases. Additionally, PATIKA uses the notion of compound graphs to represent abstractions, which are logical groupings that may be used to handle the complex and incomplete nature of the data. Figure 3 shows example pathways drawn at biological entity and state/transition levels.

More formally, a *compound pathway graph CP *= (*G*, *I*) is a 2-tuple of a *pathway graph G *and a directed acyclic *inclusion graph*. Notice that this is different than traditional compound graphs as we do not require compound structures in our pathway models to form a tree. *I*, where

• *V *(*G*): union of nodes denoting bioentities, states, transitions, molecular complexes, and abstractions of five distinct types: regular, incomplete state, incomplete transition, homology state, and homology transition;

• *E*(*G*): union of interaction edges of various types (such as PPI edges at bioentity level and activator edges between a state and a transition), some of which are directed and/or inhibitory;

• *V *(*I*) = *V *(*G*);

• *E*(*I*): union of inclusion edges for defining compound structures (molecular complexes and abstractions).

In order for a compound pathway graph *CP *= (*G*, *I*) to comply with the PATIKA ontology, it needs to satisfy certain additional invariants; for instance, regular abstractions cannot have a direct interaction (edge).

## Results

PATIKA*web *[18] is a pathway analysis tool with a distributed architecture, where the server is composed of a database component and an application server. The implementation uses the JSP (JavaServer Pages) edition of the Tom Sawyer Visualization technology [21] to handle highly-dynamic and advanced visual content along with Java™, JavaScript™ and DHTML/HTML.

The query component of PATIKA*web *was implemented as a Java applet, embracing all algorithms discussed in this paper. See Additional file 1 for a sample querying scenario comprised of a number of these operations.

### Query interface of PATIKAweb

**Figure 4:**
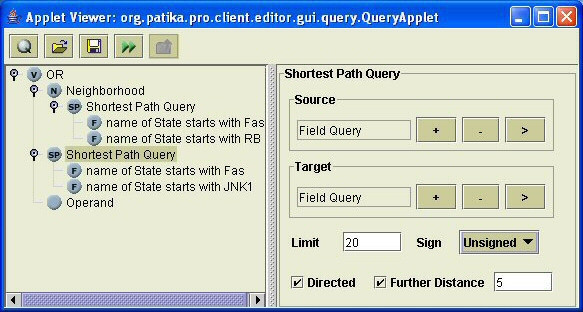
**Sample query**. Sample query tree to find the **union **of 1-neighborhood of the objects on the shortest path from states whose name starts with "Fas" to states whose name starts with "RB" **with **the shortest path from states whose name starts with "Fas" to states whose name starts with "JNK1" [18].

**Figure 5:**
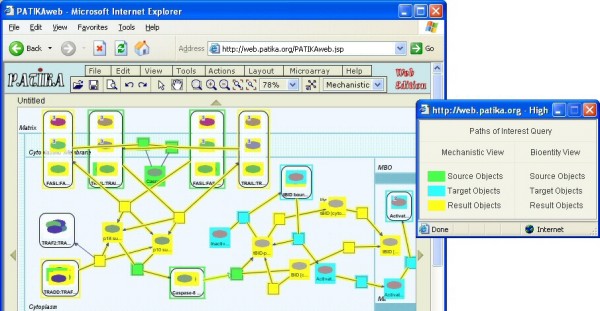
**Sample query result**. Mechanistic view of the result of the following sample query: paths-of-interest (yellow) with source of all mechanistic nodes whose names contain "caspase-8" (green) and target for those whose names contain "bax" (cyan) with limit 8; Highlight Legend Dialog for this query is shown on the right.

For utmost flexibility, the queries are allowed to be recursively organized into a tree in PATIKA*web*, in which the result of one query might be a source and/or target for another. Queries may be related to each other through "AND" and "OR" operators (Figure 4). A query may be executed not only on the database but also on the current local pathway model. When a query initiated from the query dialog finishes, the resulting pathway model is summarized by a Query Result Dialog. The model can be viewed in either or both of bioentity and mechanistic levels. Figure 5 shows a sample query result.

### Experiments

We performed a number of experiments to test our algorithms using the implementation within PATIKA*web*. The tests were performed on an ordinary personal computer using a randomly created integrated pathway knowledge base, consisting of about 20,000 pathway nodes and 30,000 edges. The knowledge base was held in memory. However, with the success of current high performance object/relational persistence and query services, the slowdown should not be dramatic when it is on disk. Our experiments revealed that our theoretical analysis is in line with the results of our implementation. Below, we provide the details.

**Figure 6:**
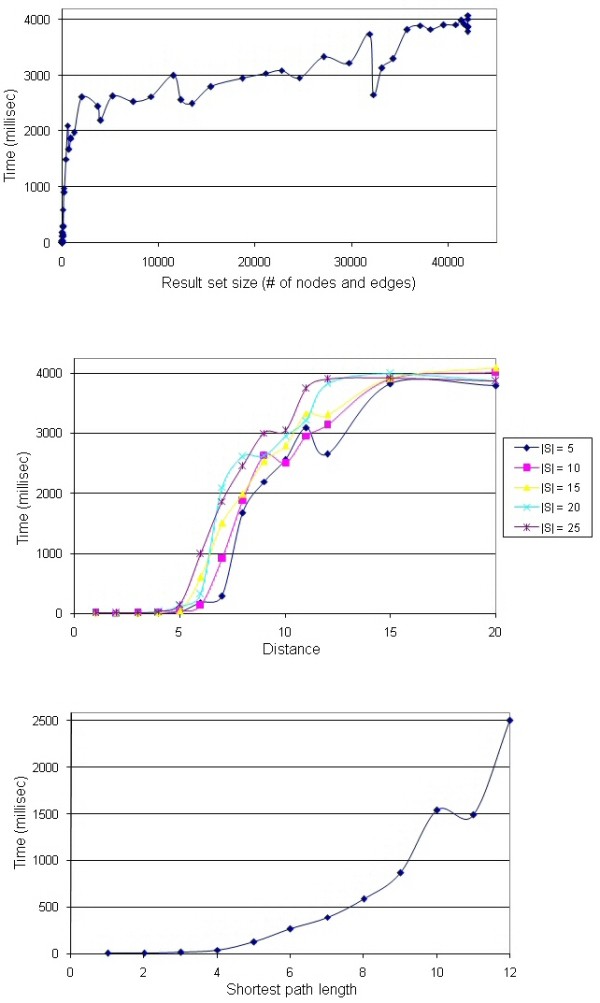
**Query parameters vs. execution time**. **(top)** Result set size (|NB(*S*, *k*)|) vs. execution time for GoI algorithm. **(middle) **Distance (*k*) vs. execution time for GoI algorithm. **(bottom) **Shortest length vs. execution time of shortest-path algorithm.

One set of experiments computes the graph of interest GoI(*S*, *k*). The time complexity of this algorithm is proportional to the number of nodes and edges in the *k*-neighborhood of nodes of interest, as Figure 6 indicates.

Experiments in this set also measure *k *versus the execution time with random source sets of various sizes. As Figure 6 illustrates, independent of the source set size, execution time increases rapidly (but not exponentially) up to a certain distance (between 12 and 15), after which it remains constant. One might expect an exponential increase in the number of nodes reached (and thus, the execution time) as the distance increases. However, in practice, up to all of the neighbors of a newly visited node might already have been visited, avoiding a combinatorial increase [22]. After a certain number of steps, all nodes are expected to have been visited.

Another set of experiments we performed was to compute the shortest paths SP(*S*, *T*, *k*, *d*). Remember that the time complexity of this algorithm is *O*(*l *+ |NB(*S*, *k*)|), where *l *is the total length of the paths enumerated. Here, *l *depends on the choice of *S *and *T*, and can be exponential in the size of the graph, in the worst case. However, our experiments show that, on average, that is not the case, and *l *is dominated by the second term. The reason for this is that, roughly speaking, if the shortest length between sets is "too long", the number of paths found is small. Similarly, if the number of paths found is large, the shortest length between sets is short. Obviously, *l *equals the number of paths found multiplied by the shortest path length between sets. Therefore, there is a tradeoff between the number of paths and the shortest length between sets. This hides the exponential behavior expected in the worst case. Figure 6 plots the shortest path length versus the execution time for the shortest path algorithm, where node sets *S *and *T *of varying sizes (chosen from set {1, 3, 5, 10, 15, 20, 25, 50}) were picked randomly. The details of this analysis may be found in Additional file 2.

## Discussion

Imagine a researcher who observes a certain gene's expression level oscillating in a manner that can be best described with a feedback loop. Figuring out which molecular interactions caused this behavior might require a review of many research articles and the integration of information across various sources. A review article or a pathway diagram can be immensely helpful, but only if it completely contains the *path *in question. In the case of a single gene, this analysis is time-consuming and painstaking. When using high-throughput data, for which one needs to consider multiple entities in a much broader scope, it becomes virtually impossible.

To face this challenge, hundreds of pathway databases were developed [23], and efforts to form an integrated map of cellular events are underway [24]. However, we also need to be able to automatically construct "pathways" by extracting relevant portions of the underlying network, based on the biological question we are asking. Compared to static pathway diagrams, such "dynamic" queries can provide a more complete picture, and can help manage complexity by removing portions that are not of interest. The algorithms and framework presented here provide such facilities for an elementary set of biological questions.

One can extend this basic set by adding algorithms that perform advanced queries, such as flux balance analysis [25], isomorphic graph matching [6], and differential expression analysis [26]. To complement advances in pathway databases and tools, we expect algorithms such as these to become commonplace in biological research. Towards this goal, we hope to make this framework and basic set of operations available as a public library, ready to accept pathway models in standard formats such as BioPAX.

## Methods

We first explain how certain special pathway structures, namely compound structures and ubiquitous entities, are to be handled. Then, we define a number of graph-theoretic problems along with associated algorithms.

### Compound structures

All the query operations described later, make use of traversals over a pathway knowledge base to compute the desired sub-graph of interest. The traversal over pathway objects represented by some sort of a compound structure (e.g., regular abstractions or molecular complexes as defined by the PATIKA ontology) calls for a special mechanism. Take as an example a breadth-first traversal, starting with node *n*_3 _over the compound graph in Figure 2. When the traversal reaches the compound node *c*_1_, should it also visit its member nodes *n*_4 _and *n*_5_? Or if it reaches its member *n*_5_, should it also visit its sibling *n*_4_, and continue its downstream towards *n*_1_?

The answer depends on the context. If an underlying "equivalence relation" exists between a compound node and its members, a traversal reaching a node in an equivalence set should also reach and visit other nodes in this set. For instance, when a traversal reaches a gene that is a member of a homology abstraction (i.e., it is inside a compound structure along with its homologous genes), it should also be considered to have reached its siblings (i.e., its homologous genes). On the other hand, reaching a member of a regular abstraction should rarely be interpreted as reaching all the other members of that abstraction. Thus, it is best to let the user decide how the traversals over compound structures should be configured for each type of such structures.

**Figure 7:**
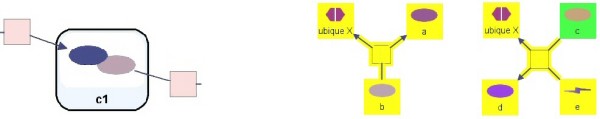
**Example traversals of complexes and ubiquitous molecules**. **(left) **The traversal reaching complex "c1" from the left transition will continue to the right transition only if the "Link Members of Complex" option is true. **(right) **Whether or not protein states "a" and "b" are in the 4-neighborhood (yellow) of state "c" (green) depends on whether traversal over ubiquitous molecules ("ubique X") is allowed. In this case, it was allowed.

This problem may be addressed by using traversal options that define how the traversal continues upon reaching a compound structure or a member of the compound structure. Note, however, that we need two flags per type of compound structure, as they might be set to be different:

• *Link a compound structure and its members*: For instance, should reaching a homology state be interpreted as also reaching its members (i.e., the genes that are homologous) and vice versa?

• *Link members of a compound structure*: For instance, should reaching a member of a molecular complex be interpreted as reaching all members of this complex (thus the traversal should be able to continue from other members as in Figure 7)?

For the ontology that we assume, there are six distinct types of compound structures: five types of abstractions (homology states and transitions, incomplete states and transitions, and regular abstractions) and molecular complexes. Once such a set of options is defined, the modification needed in a query algorithm to support compound structures is rather straightforward. When a compound node or a member of a compound is visited during a traversal, the algorithm forms a set of "equivalent" nodes, and continues the traversal from these equivalent nodes as well as from the visited node. In other words, the algorithm acts as if it's not only this node that is a neighbor of the previously visited node but also its equivalent nodes.

More formally, node *x *is called *equivalent for traversal *with node *y *if and only if

• *x *and *y *are members of the same compound node, and the user defined flag for linking members of this type of compound structure is true, or

• one is a compound and parent node (possibly an indirect parent through multiple levels of nesting) of the other, and the user defined flag for linking this type of compound structure and its members is true.

Notice that equivalence for traversal is a binary relationship that is *not *transitive.

Thus, node *x *is called a *compound neighbor *of *y *if and only if {*x*, *y*} ∈ *E *or *y *is equivalent for traversal with some node *z *with {*x*, *z*} ∈ *E*. Furthermore, a *compound path *between two nodes *n*_0 _and *n*_*k *_is a non-empty graph *P *= (*V', E'*), where

Edges outside *E *do not count towards the length of compound paths.

For instance, a breadth-first traversal over a compound graph can be sketched as follows:

**algorithm **COMPOUNDBFS(*root*)

1 *Q *:= {*root*}

2 **while ***Q *≠ ∅ **do**

3    *n*_1 _:= *Q*. DEQUEUE()

4    *N *:= *n*_1_. COMPOUNDNEIGHBORS()

5    **for ***n*_2 _∈ *N ***do**

6       **if ***n*_2_.VISITED ≠ true **then**

7          *Q*.ENQUEUE(*n*_2_)

8   //process *n*_1_

9    *n*_1_.VISITED := true

The only difference from the regular breadth-first traversal, here, is line 4. The neighbors of the currently processed node *n*_1 _are identified not only based on the downstream nodes of *n*_1_, but also on the nodes equivalent to these downstream nodes with respect to compound structures. This simple difference makes it easier to modify existing graph-theoretic algorithms to add support for compound structures.

### Ubiquitous entities

Another type of biological object that requires special attention is *ubiquitous *molecules, which typically participate in many different biological activities, have relatively constant concentration and do not transmit a signal. For instance, a ubiquitous molecule such as ATP might be involved in hundreds if not thousands of reactions at the mechanistic level. Thus, one might prefer not to link two reactions whose only common actors are these kinds of molecules. Therefore, traversal over ubiquitous molecules should also be able to be controlled by customized options. Figure 7 explains this with an example. Modification needed in graph algorithms to handle ubiquitous molecules is rather straightforward.

## Algorithms

A careful requirements analysis has yielded the following graph-theoretic problems and associated algorithms that might be useful in querying cellular pathway databases. This list is by no means exhaustive and can easily be extended. Unless otherwise stated, all algorithms have both directed and undirected versions.

### Neighborhood of entities

**Figure 8:**
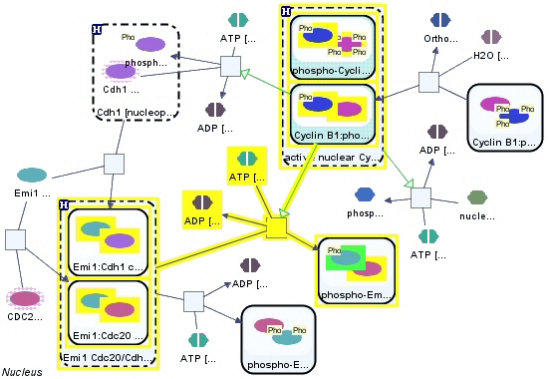
**Neighborhood**. 2-neighborhood (yellow) of phosphorylated Emi1 (Ser 182) (green) in a partial pathway in nucleus. Compound nodes with dashed borders represent homologies, whereas compound nodes with solid borders represent molecular complexes.

A simple yet powerful operation for exploring pathways is finding the neighbors of a specified source entity or node within a certain distance (Figure 8). Most pathway visualization tools [18,27,28] provide a way to expand or highlight the neighborhood of selected nodes. Pathway databases [24,29] generate on the fly views based on neighborhoods. Formally, *k*-neighborhood of a node set *S *can be defined as

### Upstream or downstream of entities

*k upstream *(*downstream*) of an entity *a *is composed of the entities on the incoming (outgoing) compound paths to *a *with length at most *k*. The positive (negative) upstream of an entity *a *is composed of the entities on the incoming compound path that activates (inhibits) (in the case of a mechanistic pathway, the preceding transition of) entity *a*. For instance, *k *positive upstream of a node *a *can be defined formally as

**Figure 9:**
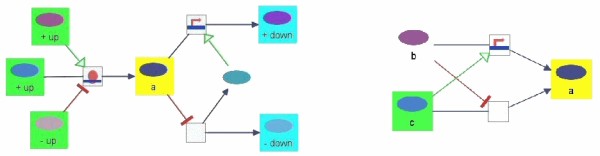
**Upstream**. **(left) **Up (green) and down (cyan) stream of protein "a" (yellow) in a partial mechanistic pathway. **(right) **Unambiguous positive upstream of node "a" (yellow) contains node "c" (green) only, as node "b" is on both positive and negative paths leading to node "a".

A node *b *might be in both the positive and negative up or downstream of another node *a*, making the effects of those streams (or associated positive and negative compound paths) ambiguous. Those nodes in the upstream (downstream) of a node *a *that lead to (are reached from) node *a *with *only *positive compound paths form the *unambiguous *positive upstream (downstream) of node *a *(Figure 9).

**algorithm **STREAM(*v*, *length*, *maxLength*, *sign*, *desiredSign*, *dir*)

1    *v*.AVAILABLE := false

2    **if ***length < maxLength ***then**

3       **for ***w *∈ *v*.COMPOUNDNEIGHBORS(*dir*) **do**

4          **if ***w *is an inhibitor **then ***sign *:= -*sign *//change sign of running path

5          **if ***sign *= *desiredSign ***then ***R *:= *R *∪ {*w*}

6          **else ***A *:= *A *∪ {*w*}

7          **if ***w*.AVAILABLE = true **then **//prevents infinite loop

8             STREAM(*v*, *length *+ 1, *maxLength*, *sign*, *desiredSign*, *dir*)

9    *v*.AVAILABLE:= true

10  **return ***R*, *A*

The algorithm performs a brute-force traversing of all the nodes in the *k*-neighborhood of the source node. It is based on a depth-first search. However, after the recursive processing of a node finishes, that node is marked as "unvisited" again, potentially leading to multiple visits of nodes and edges. More specifically, every node and edge is processed as many times as the number of distinct ways they can be reached from the source node. In other words, every possible compound path with a length limit from the source node is examined to determine if it makes a valid stream.

Naturally, the worst-case time complexity of this algorithm is exponential in the size of the *k*-neighborhood of the source node. Experiments show, however, that the execution time should be acceptable for most interactive applications for small values of *k *(for instance, up to 10).

### Common targets or regulators

There are already a number of algorithms for inferring highly connected or co-regulated subnetworks of cellular interactions and processes often called modules or pathways [30-32]. When analyzing these modules, we often want to know if there is a process or gene that is upstream of the genes in the module, which can provide a causal explanation for the co-regulation, and ultimately a way to control the module. Similarly, two pathways affecting the same mechanism in the cell is interesting since it suggests that a specific phenotype can have more than one molecular cause. For instance, Engelman et al. [33] discuss that drug resistance in lung cancer is related to an alternative pathway that leads to PI3K activation. Searching for common targets of signaling proteins can help to develop alternative treatment strategies.

Common downstream (upstream) of a source entity set *S *is the set of potential common target (regulator) entities that are in the downstream (upstream) of *all *entities in *S*. More formally, the common downstream, CD(*S*, *k*), of a source node set *S *with path length limit *k *is defined as

**Figure 10:**
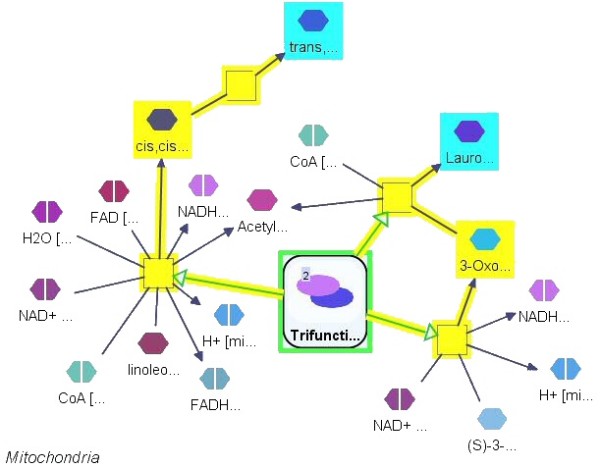
**Common regulator**. The common upstream, with a path limit of 2, of small molecules containing the word "lauro" in their name (cyan) in this partial mechanistic pathway turns out to be a single node representing a molecular complex (green). The paths from the potential common regulator to the target nodes are highlighted (yellow).

The common upstream CU(*S*, *k*) of a set *S *can be defined similarly. Figure 10 shows an example of this operation. Below is the pseudocode of this algorithm. The input parameter *dir *specifies whether we are asking for potential targets or regulators, requiring a forward or reverse BFS, respectively. Algorithm CU-COMPOUNDBFS simply increases the *reached *count of nodes in the *k*-neighborhood of seed node *n*_1_. The nodes reached during such searches are combined in a candidate set. Only the nodes in the candidate set that are reached from *all *source nodes are selected to form a result set.

**algorithm **COMMONUPSTREAM(*S*, *k*, *dir*)

1    *C *:= *R*:= ∅ //candidate and result sets, respectively

2    **for ***n*_1 _∈ *S ***do**

3       *C *:= *C *∪ CU-COMPOUNDBFS(*n*_1_, *k*, *dir*)

4    **for ***n*_2 _∈ *C ***do**

5       **if ***n*2.LABEL(*reached*) = |*S*| **then ***R *:= *R *∪ {*n*_2_}

6    **return ***R*

This operation takes *O*(|*S*|·|NB(*S*, *k*)|) time, as a BFS is performed for each node in *S*.

In addition, one might require such paths leading to potential targets or originating from potential regulators to be positive or negative. For instance, the common downstream of source nodes *S *reached by positive compound paths of length up to *k *only, denoted by CD^+^(*S*, *k*), might be of interest. However, we conjecture that the complexity of such an operation is asymptotically higher.

### Network of interest

A problem that arises frequently in high-throughput studies is gene/protein selection. For example, new high-throughput sequencing technologies enabled scanning for mutations in a large number of samples. With the current technology, the feasible number of genes that can be sequenced is in the order of tens to hundreds [34]. One way to select new genes for sequencing is to search within the vicinity of the genes that are already implied in that cancer. Particularly, genes that connect one or more of these usual suspects via a signaling path are more likely to be critical for the disease.

**Figure 11:**
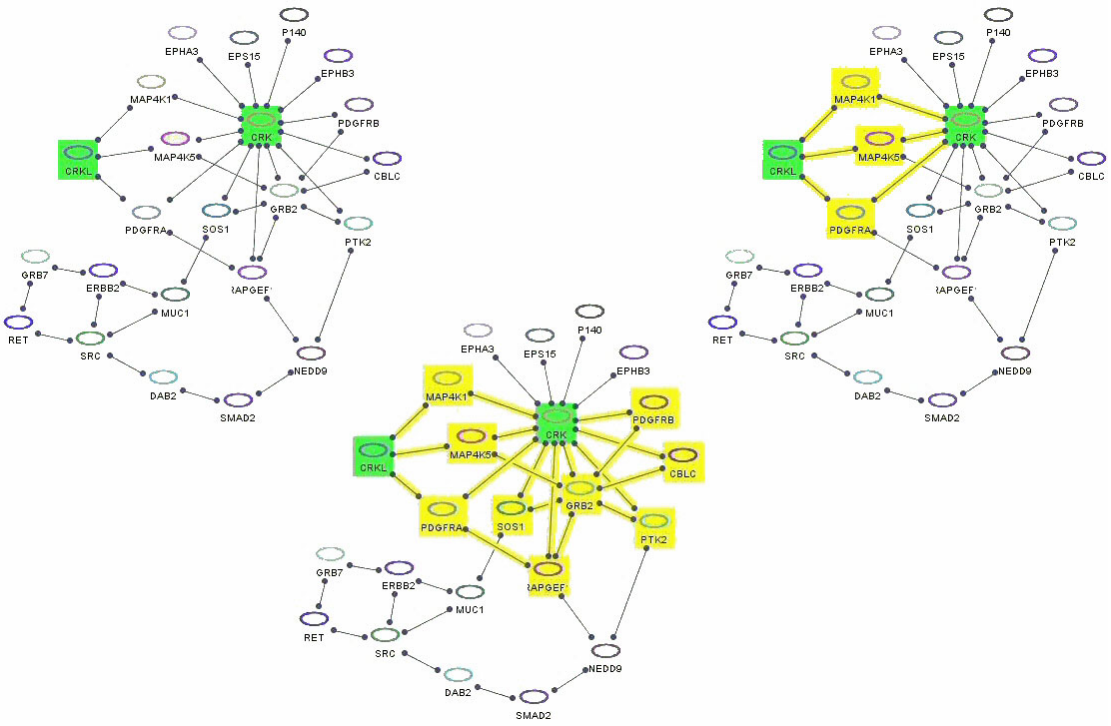
**Graph of interest**. **(left) **A PPI network with proteins of interest CRK and CRKL (green); **(middle) **Graph of interest (yellow) formed by using paths of length up to 3 (*k *= 3) between nodes of interest (green); **(right) **Graph of interest with *k *= 2 (*k *= 1 returns no results).

Given a graph *G *and a set of entities of interest *S *(e.g., genes of interest), network or graph of interest finds in *G *all compound paths of length at most *k *between any two entities of a specified entity set. The subgraph of *G *induced by the nodes of the resulting set gives the graph of interest. More formally,

As the name suggests, this operation is aimed at finding a "maximal" subgraph comprising all the nodes of interest complemented by the "missing links" among these nodes; the parameter *k *defines how long the paths, which link nodes of interest to form a graph of interest, are allowed to be. Figure 11 explains this operation with an example. Below is the pseudocode for this operation. Here, two separate BFS are to be run in forward and reverse directions, and combined to form a candidate set. The nodes in this candidate set satisfying the maximum path length constraint are put in a result set, which is "purified" by a post-processing phase, during which degree 1 nodes that do not lie on paths between source set nodes (effectively, subgraphs that are trees in the result, coinciding with the source set only at their roots) are pruned iteratively.

The following algorithm can handle directed compound pathways. It could, however, be simplified for undirected pathways by removing a redundant call to GOI-COMPOUNDBFS.

**algorithm **GRAPHOFINTEREST(*S*, *k*)

1    *C *:= GOI-COMPOUNDBFS(*S*, *k*, *fwd*) ∪ GOI-COMPOUNDBFS(*S*, *k*, *rev*)

2    **for ***q *∈ *C ***do**

3       **if ***q*.LABEL(*fwd*) + *q*.LABEL(*rev*) ≤ *k ***then ***R *:= *R *∪ {*q*}

4    *R *:= PURIFY(*S*, *R*)

5    **return ***R*

**algorithm **GOI-COMPOUNDBFS(*S*, *k*, *dir*)

1    Add all the nodes in set *S *to queue *Q*

2    Initialize *dir *labels of all the nodes in *S *to zero

3    *T*:= ∅

4    **while ***Q *≠ ∅ **do**

5       *n*_1 _:= *Q*.DEQUEUE()

6       **for ***e *∈ *n*_1_.INCIDENTEDGES(*dir*) **do**

7          **if ***dir *= *fwd ***then ***e*.LABEL(*dir*):= *n*_1_.LABEL(*dir*) +1

8          **else ***e*.LABEL(*dir*):= *n*_1_.LABEL(*dir*)

9       **for ***n*_2 _∈ *e*.COMPOUNDOTHEREND(*n*_1_) **do**

10          *T *:= *T *∪ {*e*, *n*_2_}

11          **if ***n*_2_.LABEL(*dir*) *> n*_1_.LABEL(*dir*) +1 **then**

12             *n*_2_.LABEL(*dir*) := *n*_1_.LABEL(*dir*) +1

13             **if ***n*_2_.LABEL(*dir*) *< k ***and ***n*_2 _∉ *S ***then ***Q*.ENQUEUE(*n*_2_)

14    **return ***T*

The complexity of this operation is clearly *O*(|NB(*S*, *k*)|); that is, linear in the number of nodes and edges in the *k*-neighborhood of nodes of interest.

The Paths-of-Interest (PoI) query, on the other hand, performs the same operation but in a constrained manner, *from *a specified set of source molecules *to *a specified set of target molecules. More formally,

### Shortest paths between entities

Finding shortest paths between a single or all *pairs *of vertices in a graph is a commonly used graph operation [35]. This query is a more general version of this operation, where we find and list all shortest paths between source and target sets *S *and *T*. This operation might be constrained by a parameter denoting the maximum length of such paths. In addition, a parameter for "relaxing" the shortest requirement might be useful. Thus, for instance, the shortest compound paths between two node sets *S *and *T *with maximum length *k *and "further distance" *d *can be defined formally as

**Figure 12:**
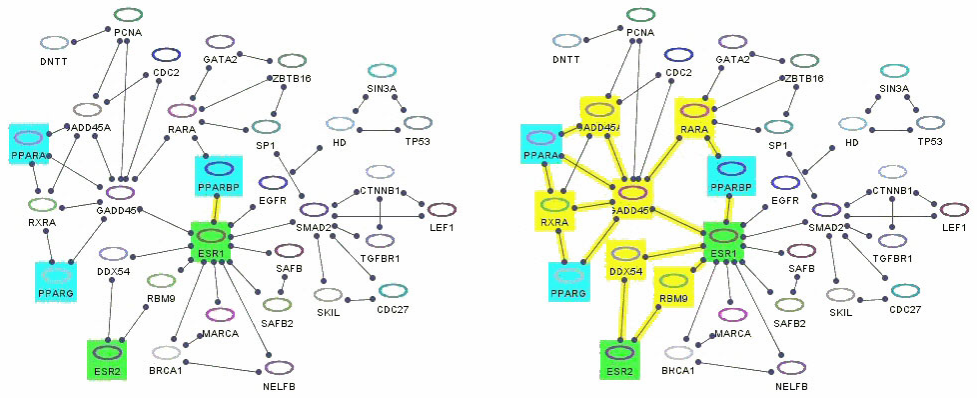
**Shortest paths**. Shortest paths (yellow) between bioentities whose names start with "PPA" (cyan) and those whose names contain "ESR" (green) with **(left) ***d *= 0 and **(right) ***d *= 2. Notice that the length of a shortest path between these two node sets is 1.

**Figure 13:**
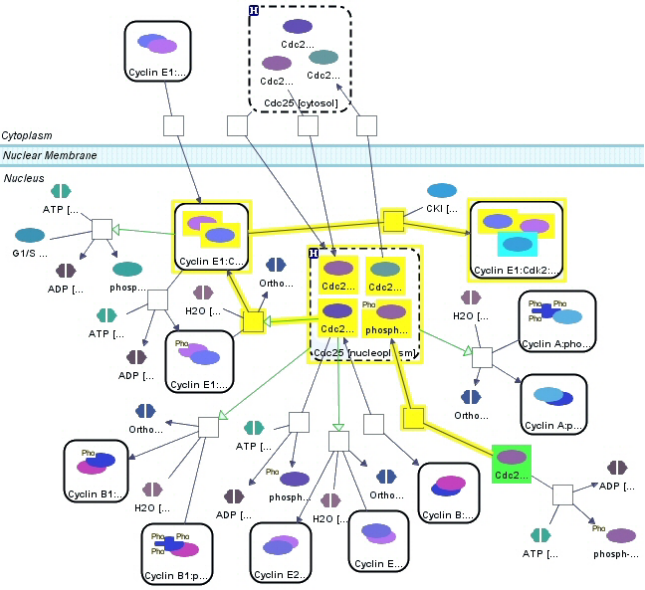
**Shortest paths**. Shortest path (yellow) between Cdc25C (green) and CKI (cyan) in nucleus. Compound nodes with dashed borders represent homologies, whereas compound nodes with solid borders represent molecular complexes.

Figures 12 and 13 illustrate this query with examples. Below, is the pseudocode for finding SP(*S*, *T*, *k*, *d*), where *mod *specifies whether edges are to be treated as directed or undirected.

**algorithm **SHORTESTPATHS(*S*, *T*, *k*, *d*, *mod*)

1    *R *:= SP-COMPOUNDBFS(*S*, *T*, *k*, *d*, *mod*)

2    **return **BUILDUPANDENUMPATHS(*S*, *T*, *R*)

**algorithm **BUILDUPANDENUMPATHS(*S*, *T*, *R*)

1    **for ***n *∈ *R ***do**

2       Construct a new path *p *for *n*

3    Add all nodes in set *R *to queue *Q*

4    *W*:= ∅

5    **while ***Q *≠ ∅ **do**

6       *n*_1 _:= *Q*.DEQUEUE()

7       **for ***n*_2 _∈ *n*_1_.COMPOUNDNEIGHBORS(*mod*) **do**

8          **if ***n*_2_.LABEL() = *n*_1_.LABEL()-1 **then**

9             *W *:= *W *∪ {(*n*_1_, *n*_2_) }

10             **if ***n*_2 _∉ *W ***and ***n*_2_.LABEL() ≠ 0 **then**

11                *W *:= *W *∪ {*n*_2_}

12                *Q*.ENQUEUE(*n*_2_)

13                **if ***n*_2 _is the first neighbor **then **Concatenate *n*_2 _to paths of *n*_1_

14                **else **Clone all the paths of *n*_1 _and add them to paths of *n*_2_

15                Update path list of *n*_2_

16                Update sign of paths of *n*_2 _with edge (*n*_1_, *n*_2_)

17    **return ***W*, paths

Here, SP-COMPOUNDBFS runs a BFS, starting with nodes in set *S *in provided *mod*, up to maximum depth *k *or shortest compound path length plus *d*, whichever is smaller, and returns the reached nodes in *T *while labeling each node with its distance from source. The overall complexity of the algorithm is *O*(*l *+ |NB(*S*, *k*)|), where *l *is the total length of the paths enumerated. Here, *l *can be exponential in the size of the graph, in the worst case. Notice that the above algorithm *enumerates *all shortest compound paths. If it suffces to find all the nodes and edges on such paths, rather than listing individual paths, BUILDUPANDENUMPATHS may be simplified, resulting in a theoretically faster operation.

In the context of pathways, one might also be interested in only positive, SP^+^(*S*, *T*, *k*, *d*), or negative shortest compound *A*-*B *paths in a given pathway graph. Another useful type of operation is to find *first k *shortest compound paths (not necessarily unique!) between specified node sets. More formally,

Where

### Feedback of an entity

Inferring causal relationships between biological entities [36,37] is both critically important and difficult. One problem stems from feedback loops abundant in biological systems. When analyzing the results of these algorithms or methods, one often wants to check if there exists a known feedback loop to flag the inferences that are potentially false.

This operation results in a list of positive or negative compound cycles that contain a specified entity. It is useful for finding feedback signals and metabolic cycles in a network. Positive feedback of a node *s *with maximum length *k *is defined as

**Figure 14:**
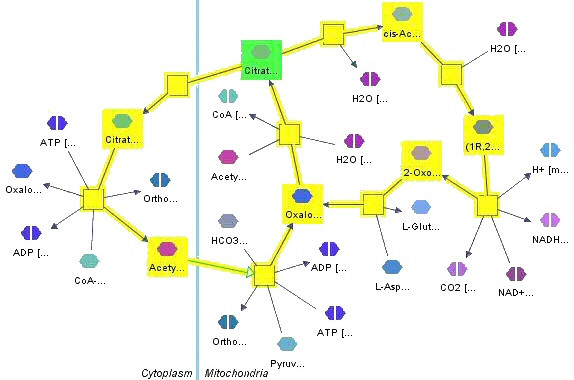
**Positive feedback**. Positive feedback (yellow) of a specified Citrate state in mitochondria (green) with up to length 10. The result contains two metabolic cycles; one in mitochondria (of length 10) and one through cytoplasm (of length 8).

Figure 14 illustrates this with an example. Our algorithm is based on generating all cycles starting from a given set of source nodes in a directed graph, as described in [38].

The algorithm starts at source *s*, and builds a directed path *sn*_1_*n*_2_*n*_3_⋯*n*_*k *_in a depth-first manner. A cycle is found if the next vertex *n*_*k*+1 _equals *s*. After generating this cycle, the next edge going out of *n*_*k *_is explored. If all edges going out from *n*_*k *_have been explored or the maximum length is exceeded, the algorithm backs up to the previous vertex *n*_*k*-1_, and continues. This process continues until we try to back up past the source node *s*. At that point, all cycles involving *s *have been discovered, so *s *can be removed from the graph, and the process can be repeated until the source set becomes empty.

To prevent traversing cycles originating at a vertex *n*_*i *_during the search rooted at *s*, all vertices on the current path are marked as "unavailable" extensions of that path. For this, a flag is maintained, which is set to false as soon as *n *is appended to the current path. That node will remain unavailable until the algorithm backs up past *n *to its previous vertex on the graph. If the current path up to *n *does not lead to a cycle, it will remain unavailable, even if the algorithm backs up past it. This prevents redundant dead-end searches. Vertex *n *will, however, be marked available if a cycle could not be found due to cycle length limit, because it is possible for a shorter path to form a cycle by going through *n*.

Similar to the basis operation given in [38], this algorithm is of *O*(|NB(*S*, *k*)|·(*c *+ 1)) time complexity, where *c *is the total number of cycles (positive and negative) discovered.

## Authors' contributions

All authors participated in the design of the framework and the algorithms. UD directed the research and development. AC implemented most algorithms. ED and OB helped with implementation. All authors read and approved the final manuscript.

## Supplementary Material

Additional file 1 **A querying scenario**. A sample session in which subsequent queries and complexity management operations are performed to form a model that might be of use to a PATIKA*web *user. Click here for file

Additional file 2 **Detailed analysis of shortest path length versus execution time**. Illustrates the effect of source and target set sizes in execution time for shortest path query. Click here for file
