# Supplementary material for: Algorithms for effective querying of compound graph-based pathway databases
Source: BMC Bioinformatics. 2009 Nov 16;10:376. doi: 10.1186/1471-2105-10-376 (PMC2784781; doi:10.1186/1471-2105-10-376)
Supplement: Additional file 1 — A querying scenario. A sample session in which subsequent queries and complexity management operations are performed to form a model that might be of use to a PATIKAweb user. [file 1471-2105-10-376-S1.PDF]

Additional file 1: A querying scenario

Following is a sample session in which subsequent queries and complexity management operations are performed to form a model that might be of use to a PATIKAweb user.

Suppose the user is studying the effects of FAS Ligand on apoptosis. One good way to start is by searching for the relations between FAS Ligand and the Caspase complexes in the cell.

In order to find out the states of FAS Ligand in the cell, we perform the query in Figure 6, where we ask for simple states whose names start with "FASL".

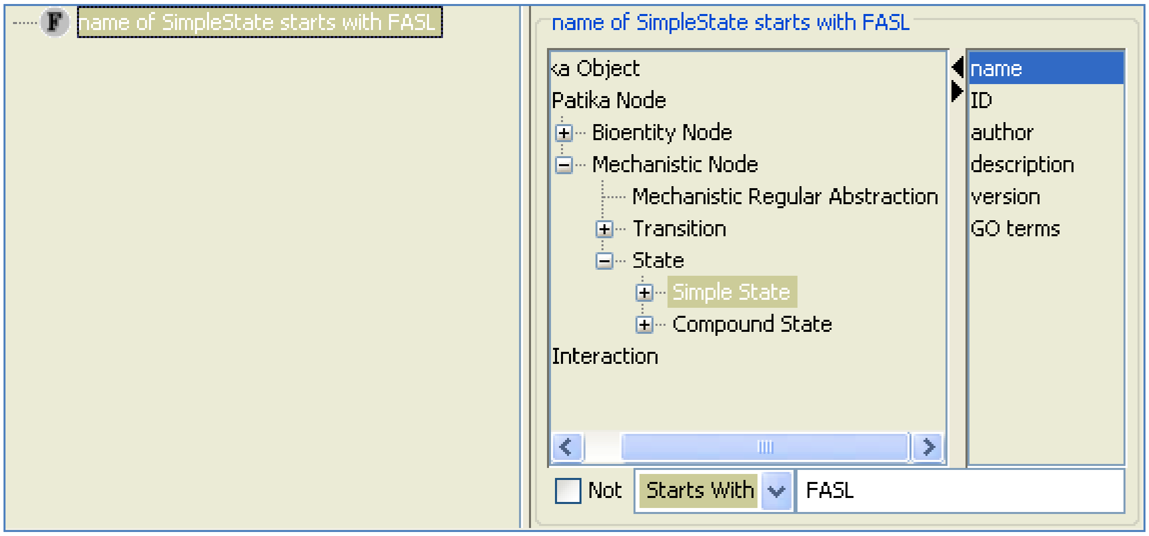

Figure 1. Query for simple states whose name starts with "FASL".

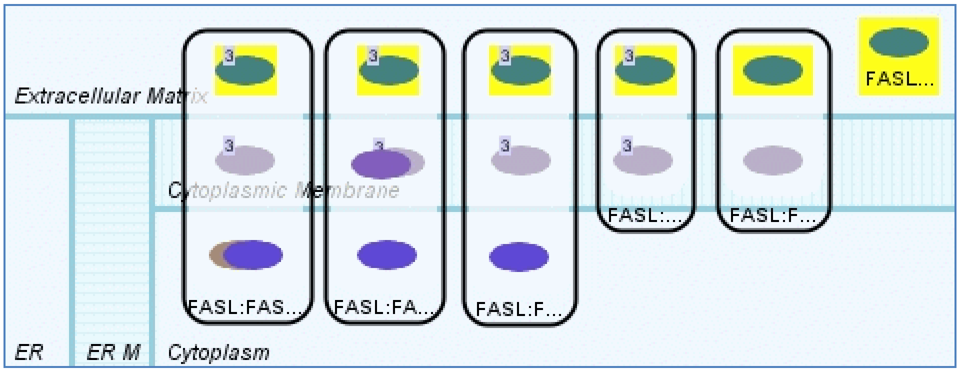

Figure 2. Result (yellow) of the FAS Ligand query in Figure 6

We see 6 states highlighted in the result of the query (Figure 7). One is the free extracellular FAS Ligand, and remaining ones are members of several complexes spanning the cytoplasmic membrane.

And we may check how many Caspase complexes we have in the database, which are not a precursor or a pro-caspase (Figure 8).

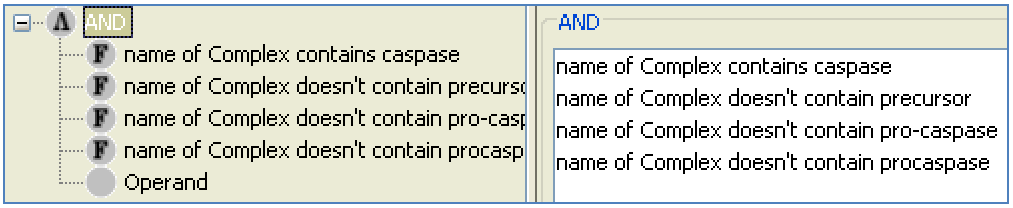

Figure 3. Query for Caspase complexes, which doesn't contain words "precursor", "pro-caspase" or "procaspase".

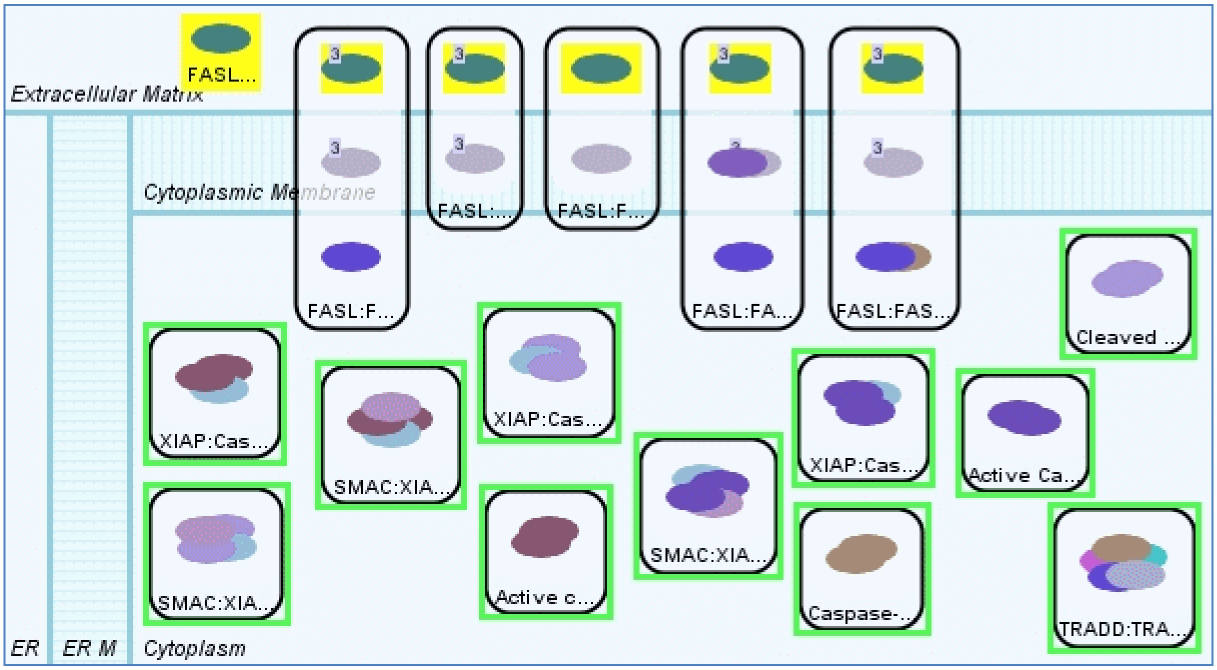

Figure 4. Result (green) of Caspase query in Figure 8 added to the existing model.

Caspase query returns a total of 11 complex molecules, which are all in cytoplasm (Figure 9). Now we know that the database contents that we want to "start from" and we want to "reach to". The most popular query for finding relatively short paths between source and target molecules is the "Shortest Path Query". We may use the previous FAS Ligand and Caspase field queries as the source and target fields of the shortest path query (Figure 10).

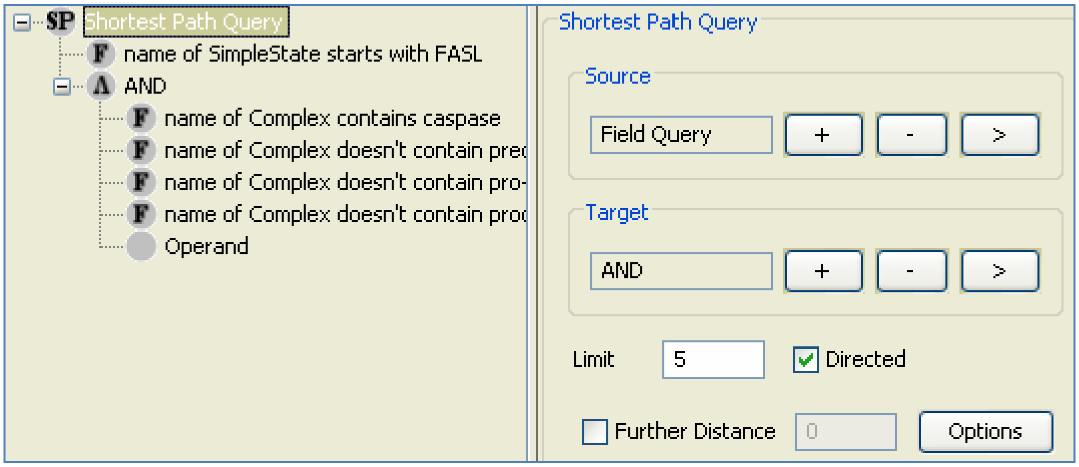

Figure 5. Shortest path query using the previous queries as source and target; the query limits the distance to 5 and considers directions.

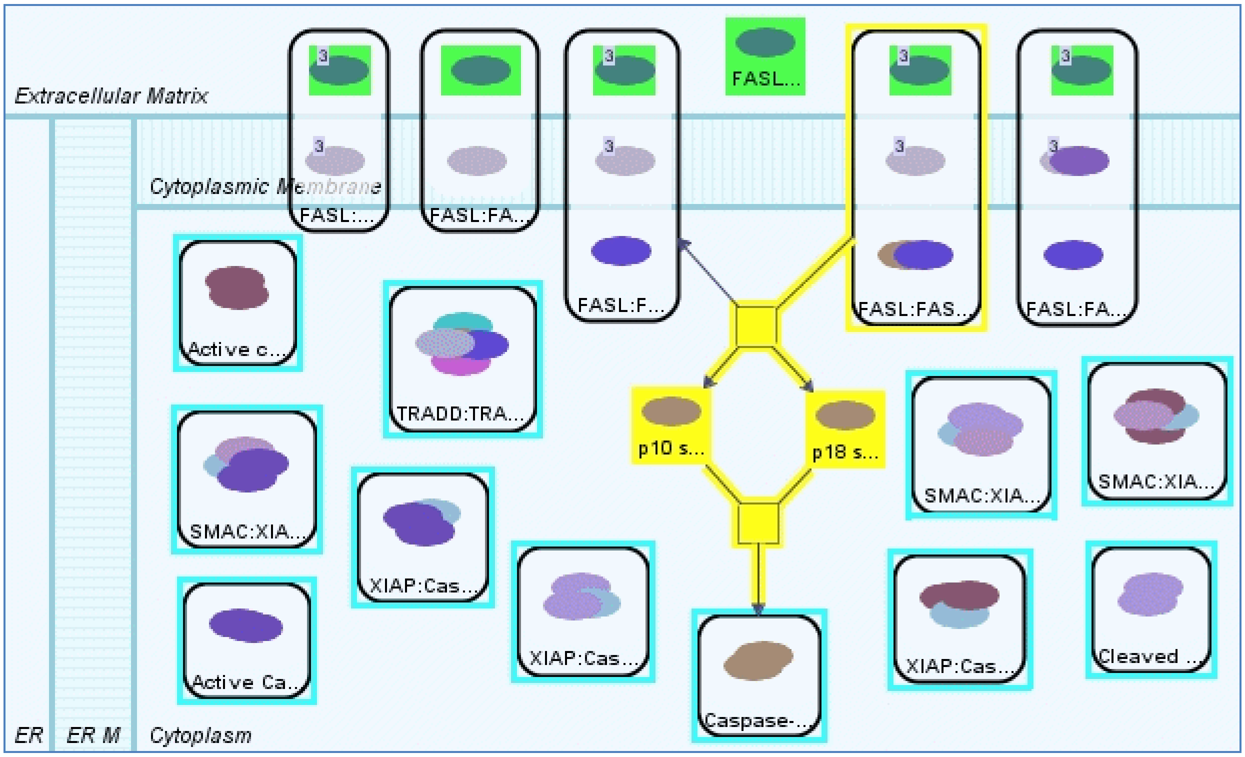

Figure 6. Result of the shortest path query in Figure 10; we find that the two shortest paths (yellow) from FAS Ligand (green) to Caspase complexes (cyan) goes to Caspase-8 dimer in cytoplasm, each with 2 transitions (4 steps).

Result of the shortest path query retrieves paths of length 4 (Figure 11). These are paths involving the FAS Ligand complex on the cytoplasmic membrane and the Caspase-8 dimer in cytoplasm. This picture might be very helpful but it still has many missing relations.

There are several ways to obtain a more complete picture. First alternative is to use the shortest path query with the "Further Distance" parameter. Figure 12 shows the same query with further distance set to 8. Since the shortest path length is 4, this query would bring us the paths from source to target nodes of length at most 12. Figure 13 shows the resulting model.

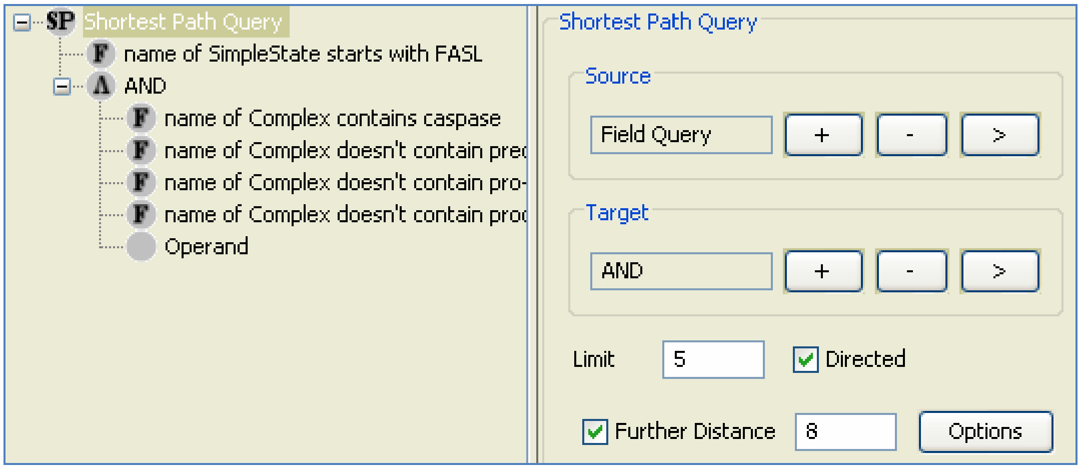

Figure 7. Shortest path query with further distance set to 8.

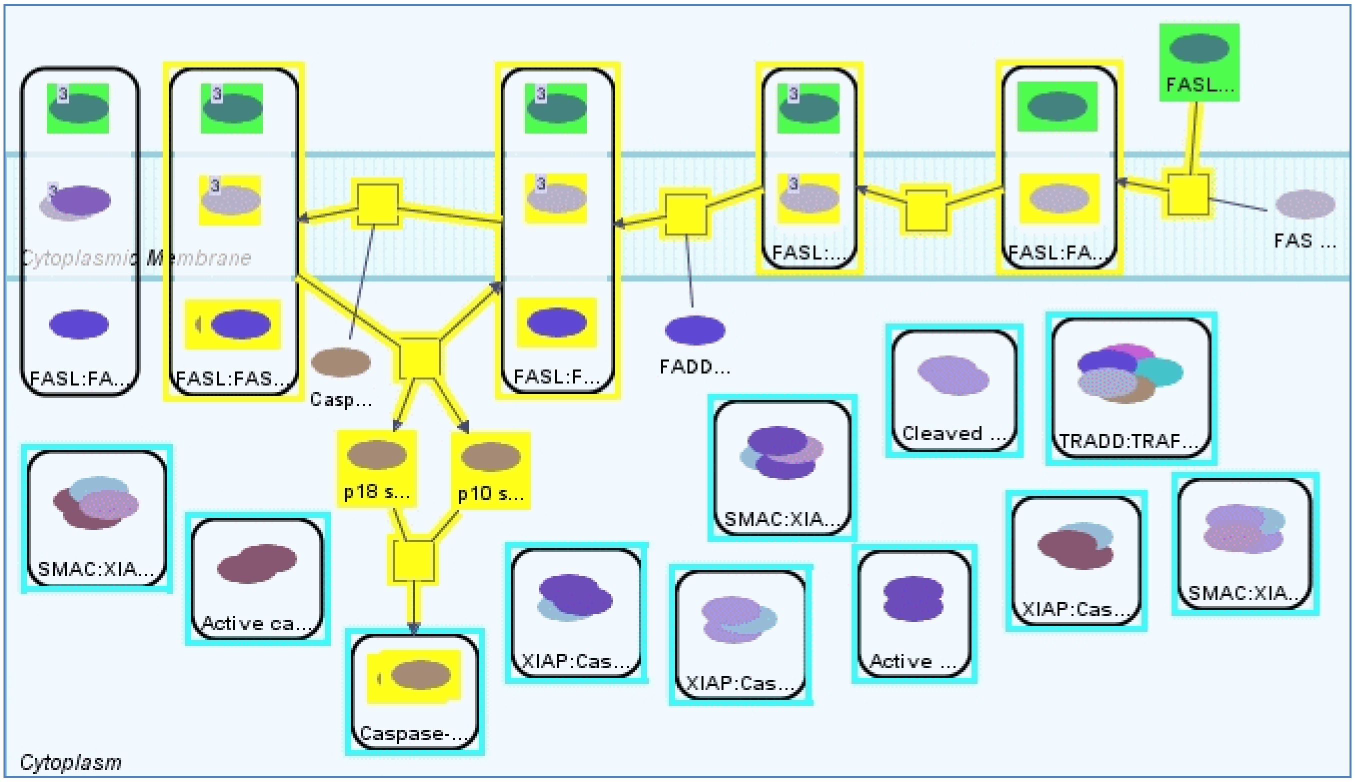

Figure 8. Result of the shortest path query in Figure 12. Paths of length up to 12 (yellow) are found between source (green) and target (cyan) sets, since the shortest path length is 4.

Another way of doing the same query is to use a “Paths-of-Interest” (PoI) query with limit 12. Since this query will bring all paths of length at most 12, between source and target sets, the result will be identical to the previous shortest path query with further distance 8. Thus PoI query is simply a more convenient way of querying paths when we have a good estimation of the length of the shortest path.

When finding paths between source and target sets is not sufficient, the user has the option to do a “Graph-of-Interest” (GoI) query. GoI query aims at completing the “missing links” (and molecules on these links) among a set of molecules of interest that is no longer than a specified limit. So a “minimal”

graph including the specified objects of interest can be constructed through this query. Figure 14 shows a directed GoI query with limit 5, where the previous source and target sets are joined into an OR query as molecules of our interest.

Since the GoI query finds all paths between a number of seed nodes (not from a specified source to specified target), the result contains more paths, not necessarily depicting a direction in the information flow. In the resulting model (Figure 15) we see that there are two isolated components. First one contains the previous FAS Ligand path we have found. We see that an additional Caspase complex is connected; however, the graph does not imply that this new Caspase complex has been involved in the FAS Ligand signaling process. Second component contains all other Caspase complexes. Notice that only two Caspase complexes have a relation with FAS Ligand signaling process in the database (at least within the distance we have specified); the user may choose to concentrate on these for further analysis.

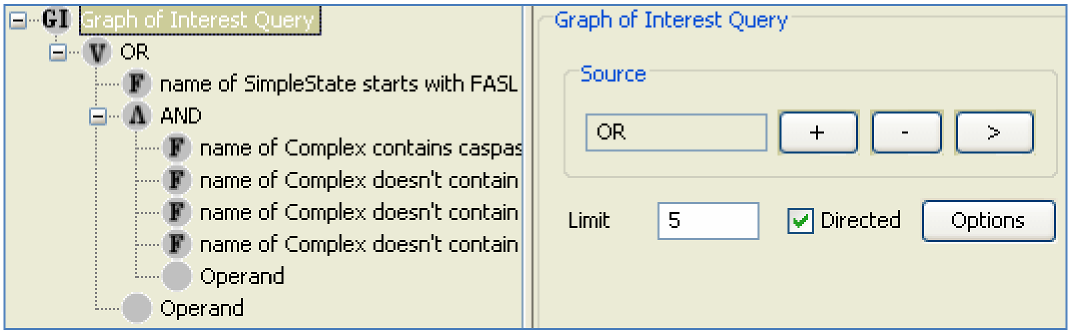

Figure 9. A GoI query where the previous FAS Ligand and Caspase complex queries are gathered into an OR query and used as seed (molecules of interest).

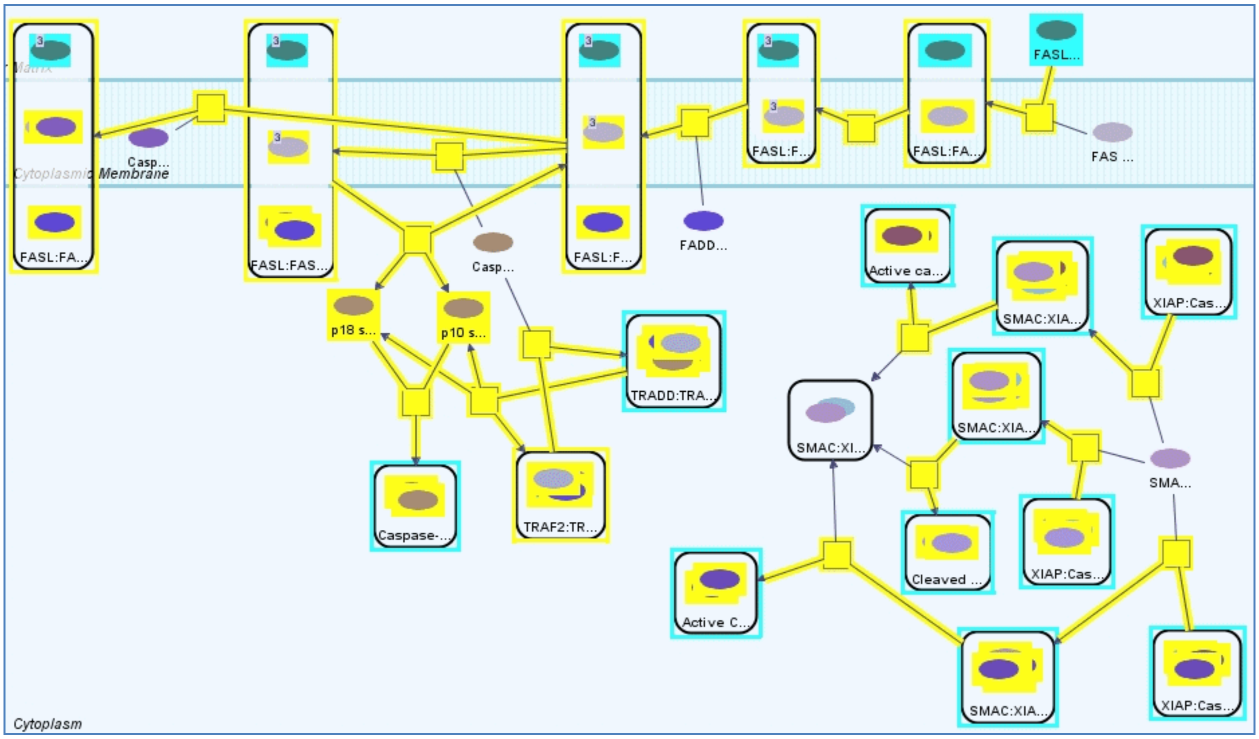

Figure 10. Result of the GoI query in Figure 9.

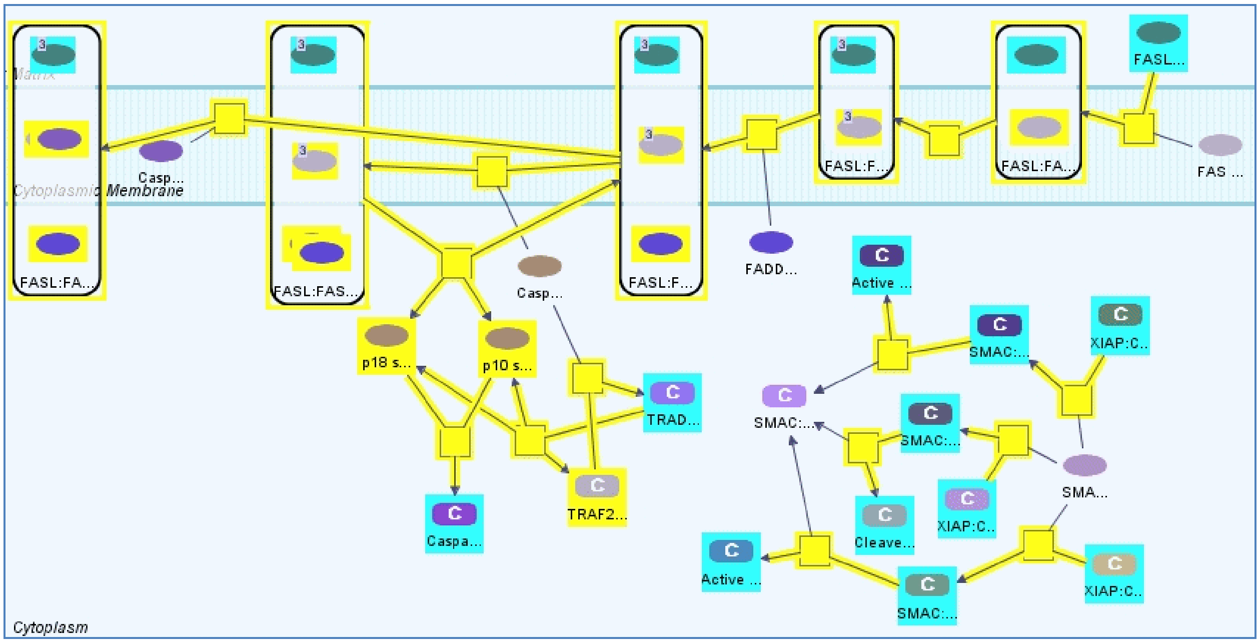

Figure 11. Result of the GoI query in Figure 9 after single-compartment complexes are collapsed for a simpler view.
