# Supplementary material for: Algorithms for effective querying of compound graph-based pathway databases
Source: BMC Bioinformatics. 2009 Nov 16;10:376. doi: 10.1186/1471-2105-10-376 (PMC2784781; doi:10.1186/1471-2105-10-376)
Supplement: Additional file 2 — Detailed analysis of shortest path length versus execution time. Illustrates the effect of source and target set sizes in execution time for shortest path query. [file 1471-2105-10-376-S2.PDF]

Additional file 2: Detailed analysis of shortest path length versus execution time

Figure 1 illustrates the effect of source and target set sizes in execution time for shortest path query. Varying set sizes from the set {1, 3, 5, 10, 15, 20, 25, 50} were taken as input to the shortest path query yielding potentially different shortest path lengths. Notice that shortest path lengths obtained for a fixed source/target set size are within a close range.

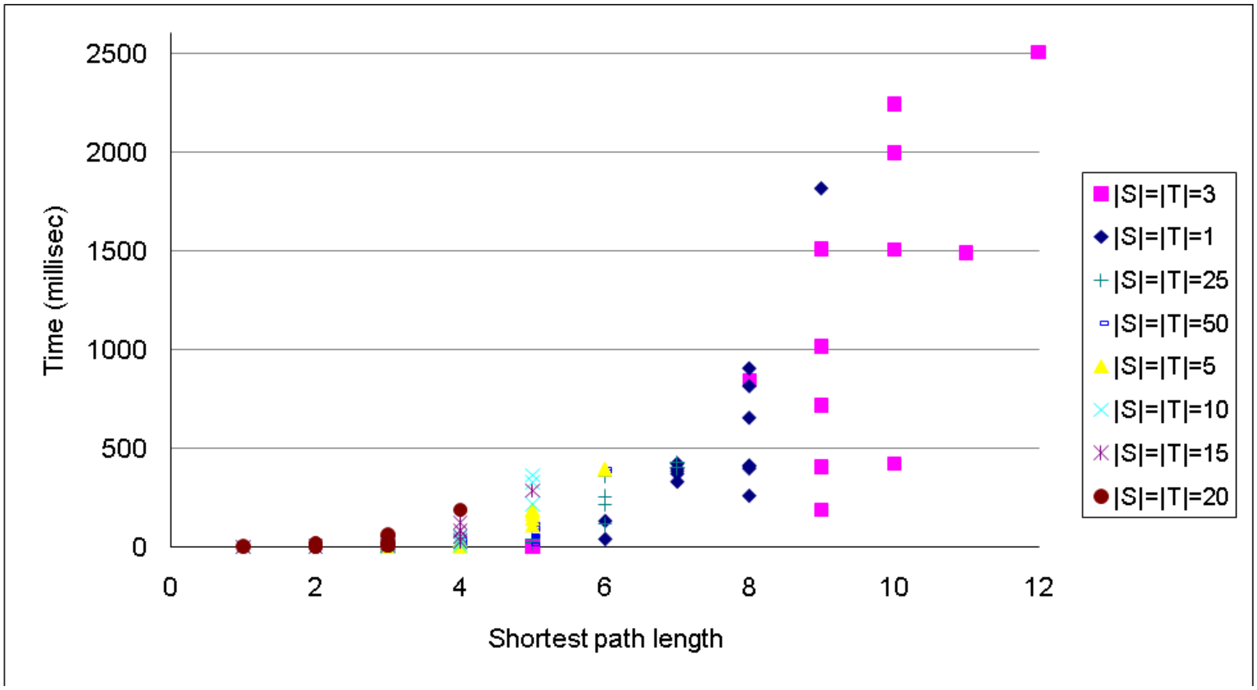

Figure 1. Shortest path length versus execution time for varying source and target node set sizes.
